# Engagement of TREM2 by a novel monoclonal antibody induces activation of microglia and improves cognitive function in Alzheimer’s disease models

**DOI:** 10.1186/s12974-020-01980-5

**Published:** 2021-01-09

**Authors:** Michael Fassler, Maya Saban Rappaport, Clara Benaim Cuño, Jacob George

**Affiliations:** Kaplan Medical Center, 1 Pasternak St, 76100 Rehovot, Israel

**Keywords:** Neuroinflammation, TREM2, Alzheimer’s disease, Mouse model, Monoclonal antibody

## Abstract

**Background:**

Genetic variants and mutations in triggering receptor expressed in myeloid cells (TREM2) are associated with premature and late onset Alzheimer’s disease (AD).

**Methods:**

We developed a panel of monoclonal antibodies, the selected lead of which was avidly shown to bind the extracellular domain of human and murine TREM2.

**Results:**

By engaging membrane-bound TREM2, the selected antibody was shown to promote their cellular proliferation, uptake of oligomeric beta amyloid/apoptotic neurons, and activation in a Syk and Akt dependent manner. The antibody was shown to avidly bind soluble TREM2 in the CSF from AD patients and blunted the proinflammatory program driven by its intracerebral injection. Upon in vivo treatment, the antibody was shown to improve cognitive function in experimental amyloidopathy models and to facilitate plaque-associated microglial coverage and activation.

**Conclusion:**

Thus, we describe a novel monoclonal antibody targeting membrane bound and soluble TREM2, that improves cognitive function by inducing microglial activation and attenuating chronic neuroinflammation.

## Introduction

Alzheimer’s disease (AD), the most common form of dementia, is evident histopathologically by the abnormal accumulation of amyloid plaques, hyperphosphorylated tau aggregates, and microgliosis [21]. TREM2 has been shown to be involved in neuroinflammation and in the metabolic fitness, proliferation, survival, and phagocytic capacity of microglia [19].

Gene network analyses of human AD brains and mouse models of AD have highlighted a central role for microglia in AD and, in particular, TREM2 and its binding partner TYRO protein tyrosine kinase-binding protein (TYROBP), also known as DNAX-activation protein 12 (DAP12) DAP12/TYROBP [11]. Whereas heterozygous variants in TREM2 are associated with AD [2, 3, 8], homozygous variants in TREM2 or its binding partner DAP12/TYROBP cause polycystic lipomembranous osteodysplasia with sclerosing leukoencephalopathy (PLOSL), also known as Nasu-Hakola disease (NHD). NHD is a rare autosomal-recessive early-onset dementia characterized by behavioral changes and cognitive decline, with or without pathological bone fractures [2, 3, 13].

The mechanism by which TREM2 influences to neurodegeneration remains obscure. Furthermore, studies investigating the impact of TREM2 signaling on the inflammatory response have produced contradictory results, demonstrating either an anti-inflammatory or a pro-inflammatory role for TREM2 [4, 6, 7, 15, 18]. Recent studies have identified a role for TREM2 in microglial survival [22], as well in regulating energy metabolism [20]. Conflicting data has been reported on the role TREM2 in phagocytosis [5, 9, 10, 17, 22, 23] in humans potentially related to interspecies differences [16].

Herein, we developed a panel of agonistic and antagonistic mAbs, binding the extracellular domain of TREM2 the selected antibody of which was shown to be capable of activating microglia expressing TREM2 thereby facilitating uptake of oligomeric beta amyloid and attenuating cognitive decline in amyloidopathy models of Alzheimer’s disease but would also be relevant to all neurodegenerative diseases.

## Methods

### Subjects and tissue sampling

This study was performed at Kaplan Medical Centre in Rehovot, Israel, under appropriate Institutional Review Board approval. Blood samples were obtained from the Israel National Blood Services except for CSF and brain specimens included in the study which were collected at the Cambridge Brain Bank supported by the NIHR Cambridge Biomedical Research Centre.

### Animals

5xFAD animals were bred in house (mutations were analyzed using specific primers for PCR genotyping). All housing, breeding, and procedures have been reviewed and approved by “The Israel Board for Animal Experiments” and in compliance with “The Israel Animal Welfare Act.” Animals were housed under standard laboratory conditions, air conditioned, and filtered (HEPA F6/6) with adequate fresh air supply (minimum 15 air changes/hour). Animals were kept in a climate-controlled environment. Temperatures range was 20–24 °C and relative humidity range was 30–70% with a 12-h light and 12-h dark cycle.

### Generation of monoclonal antibodies

Several clones of monoclonal antibodies (mAbs) were produced according to standard protocols by Balb/C mice immunization with extracellular domain of human TREM2 protein followed by three additional boosts. After confirming the presence of polyclonal anti-TREM2 antibodies in the sera, mice were sacrificed. Cells were isolated from their spleens and hybridized with an SP2/0 myeloma line, followed by clonal screening for binding to human TREM2. The hybridomas were then grown in serum-free media for 2–3 weeks, and media were collected and concentrated by 30 kDa centricons (Biological Industries, Israel).

### Humanization of Ab-T1

Sequencing of mouse IgG was performed by whole transcriptome shotgun sequencing. Antibody sequences were analyzed for specific liabilities based on published protein motifs using an in-house system build in Microsoft Excel.

### Dot blot for Ab-T1 binding assessment

Cell lysates (HEK293T transfected with human or mouse TREM2, and Naïve HEK293T as control) were prepared in hypotonic buffer (0.01 M Tris, pH 7, 1 mM EDTA, 1 mM EGTA) freshly supplemented in protease inhibitor cocktail (P8340, Sigma), incubated on ice for 30 min, and pellet resuspended in STE lysis buffer (150 mM NaCl, 50 mM Tis-HCl, pH 7.6, 2 mM EDTA, 1% Triton-X 100).

Two micrograms (reduced conditions) of lysate samples were loaded into 0.2 μm nitrocellulose membranes (WhatmanProtan BA83, Cat No. 10401380) until dry. Samples incubated at 4 °C for 12 h with 1 μg/ml mouse Ab-T1, Ab-T2, Ab-T3, Ab-T4, Ab-T5, mouse IgG (negative control) or anti-TREM2 (Abnova MAB2056, 1:1000, positive control) as primary antibodies diluted in 1% BSA/TBST followed by goat anti-mouse conjugated to HRP (115-035-003, Jackson, 1:10,000) for additional 1-h incubation at room temperature. Membranes were developed using Fusion Solo 7S imager system (Vilber, France).

### Affinity testing of Ab-T1

Ab-T1 clone was selected and tested for its high affinity to extracellular domain of human and mouse TREM2 in surface plasmon resonance (Biacore SPR system, GE Healthcare Life Sciences), whereby anti-TREM2 antibodies are immobilized on the chip surface through an anti-mouse capture antibody (BR100838, GE), for example, a CM5 sensor chip and the human TREM2 and mouse TREM2 (SI 50149-M02H, Sino Biological) and TREM1 antigens are then run over the chip.

### Transfection of hTREM2 and mTREM2 into HEK293T cells

Naïve HEK293T cells were transiently transfected with constructs expressing human (HG11084-ACG, Sino Biological) and mouse (MG50149-ACG, Sino Biological) TREM2 (fused at the C-terminus with GFP) using JetPrime-mediated transfection (JetPrime, Polyplus-transfection) following manufacturer protocol. Cells transfected with pCDNA-GFP (without an insert gene) were used as sham control. Transfected cells were validated by flow cytometry using mouse Ab-T1 conjugated to Alexa 488 Fluor (Invitrogen, Cat No. A10235). FL1–blue laser (488 nm) was used to measure TREM2 levels indicated as relative geomean fluorescence intensity (gMFI).

### Stable cell line generation of U937 cells expressing human TREM2

Naïve U937 cells (CRL-1593.2, ATCC) were transduced with lentiviral vector carrying human TREM2 gene. Single clone was screened from cell pool generated by limited dilution and positive clones were selectively expanded.

### Immunofluorescence staining in cell culture

Transfected and Naïve HEK293T cells (HEK293T-hTREM2-GFP / HEK293T) were seeded (0.2 × 10^6^/well in a 12-well plate) on cover slips coated with 0.1 mg/ml poly-l-lysine (Sigma) and blocked in 20 mM HBSS (Gibco) supplemented with 10% FBS for 45 min at room temperature. After 24 h at 37 °C, cells were incubated with 0.5 and 1 μg/ml mouse Ab-T1 conjugated to Cy5 (mAb-T1-Cy5) as primary antibody (diluted in above blocking buffer) for 1 h at room temperature followed by additional 1 h at 4 °C. Control experiment used isotype control mouse IgG as the primary antibody (K Isotype control mouse IgG Cy5, Cat# 400115, Biolegend). Cells were rinsed twice with ice-cold PBS and fixed in 4% v/v paraformaldehyde for 12 min at room temperature. Cells on cover slips were washed 3 times in ice-cold PBS and laid down on a slide with a drop of mounting buffer (DAPI Fluoromount-G, SouthernBiotech) before visualized on a confocal laser scanning microscope (TCS SP8, Leica).

### Western blotting for TREM2 detection

Thirty to twenty-five micrograms of cell lysate/supernatants/CSF was loaded in each well for SDS-PAGE protein separation (reduced conditions) on Nuphage 4–12% gels (ThermoFisher Scientific, Cat No. NP0322BOX). Samples were transferred to 0.2 μm nitrocellulose membranes (WhatmanProtan BA83, Cat No. 10401380) and incubated at 4 °C for 12 h with mouse Ab-T1 (5 μg/ml) or mouse IgG (5 μg/ml control) as primary antibodies diluted in 1% BSA/TBST followed by goat anti mouse conjugated to HRP (Jackson, 1:10,000) for additional 1h incubation at room temperature. Membranes were developed using Fusion Solo 7S imager system (Vilber, France).

### Immunohistochemistry

Human brain tissue sections from Alzheimer disease patients and 5xFAD mice were post-fixed in 4% formalin for 48 h, dehydrated in ethanol, and embedded in paraffin. A series of sections was stained for either mouse Ab-T1, anti-Iba-1 (Abcam, ab178847), anti-beta amyloid (Abcam, ab2539)/(Biolegend, 800702 (clone 4G8)), or mouse IgG (Abcam, ab37355).

Immunohistochemical staining was performed on 4-μm sections using the Leica Bond III system (Leica Biosystems Newcastle Ltd, UK). Tissues were pretreated with epitope-retrieval solutions (ER, Leica Biosystems Newcastle Ltd, UK) followed by 30-min incubation with primary antibodies mouse Ab-T1 (2.85 μg/ml), anti Iba-1 (1:1500), anti-beta amyloid (1:600), and mouse IgG (1 μg/ml). The Leica Refine-HRP kit (Leica Biosystems Newcastle Ltd, UK) was used for detection followed by counter-stain with Hematoxylin.

### Immunofluorescentic staining of brain tissues

Slices were rinsed in 0.3% Triton X-100/PBS (3 × 5 min). In order to prevent non-specific binding, slices were pre-incubated with blocking buffer (1% bovine serum albumin (BSA) and 0.3% Triton X-100 in PBS) for 2 h at room temperature. Slices were incubated with Iba1 antibody (WAKO, 019-19741; 1:1000), mouse Ab-T1 (0.5 μg/ml), or anti CD68 (Santa Cruz Biotechnology, sc-20060 AF488; 1:200) overnight at 4 °C. Slides were rinsed with 0.3% Triton X-100 in PBS (3 × 5 min) followed by incubation with Cy5 goat anti-Rabbit IgG (Abcam, ab6564; 1:200) or Alexa Fluor 488 anti-Mouse IgG (Abcam, ab150113; 1:200) in blocking buffer for additional 2 h at room temperature. Slides were washed 3 times in 0.3% Triton X-100/PBS and a drop of mounting buffer (DAPI Fluoromount-G. Cat#: 0100-20, SouthernBiotech) was added before visualized on a confocal laser scanning microscope (TCS SP8, Leica).

### Isolation and culture of bone marrow derived macrophages

Bone marrow-derived macrophages were harvested from c57BL/6 mice and cultured in DMEM/F-12 medium. Cells were centrifuge at 500×*g* at room temperature, resuspended in 10 ml medium, counted and adjusted to a concentration of 4 × 10^6^/ml in macrophage complete medium (DMEM/F-12 , 10% FBS, pen/strep, l-glu, +M-CSF 0.1 nM). Cells were seeded (4 × 10^5^ per 10 ml petri dish) in 10 ml macrophage complete medium and incubated at 37 °C, 5% CO_2_. On day 3, 4 ml of complete medium were added to each dish and cells were grown for additional 4 days (total of 7 days) before use.

### Isolation and culture of human peripheral blood mononuclear cells

Whole blood was diluted 1:1 ratio with PBS (Ca/Mg free), gently laid in density gradient medium (lymphoprep—Cat#: 04-03-9391/01, Stemcell technologies, ratio 1:2), and centrifuged for 30 min, 1500 rpm. Buffy coat was collected into a new tube, washed with PBS, and centrifuged for 15 min in 1200 rpm. Additional wash with PBS was supplemented, and blood was centrifuged for 15 min in 1200 rpm before cells were resuspended in 10 ml PBS for counting.

### Macrophage cell culture differentiated from PBMC’s

Primary human macrophages were differentiated from peripheral blood monocytes cells (PBMC). After isolation of PBMC’s in a density gradient medium, cells were split into 12-well culture plates (Greiner CELLSTAR multiwell culture plates, 0.4 × 10^6^ cells/well) and incubated at 37 °C, 5% v/v CO2 and 95% v/v O_2_ for 1 h in a free serum medium (RPMI 1640, 2 mM l-glutamine, 1% Pen/Strep, Biological Industries). Non-adherent contaminated cells were removed and remaining monocytes differentiated to human microglia like cells for 10 days with recombinant human granulocyte-macrophage colony-stimulating factor (GM-CSF, 10 ng/ml; R&D systems) in a full RPMI medium (RPMI 1640, 10% FBS, 2 mM l-glutamine, 1% Pen/Strep) and incubated at 37 °C, 5% v/v CO_2_ and 95% v/v O_2_.

### Preparation of beta amyloid oligomers

Lyophilized 1 mg of ultrapure beta amyloid (1-42) monomers (A-1167-2, rPeptide, USA) was thawed at room temperature for 10 min followed by addition of sterile DMSO to a final concentration of 5 mM. Beta amyloid was transferred to a new low-binding, sterile 1.5 ml microcentrifuge tube (Protein LoBind Tube 1.5, Eppendorf tubes, Cat no.: 022431081) followed by addition of cold phenol-free F12 cell culture media (supplemented with 146 mg/L l-Glutamine; BioSource), diluting to a final concentration of 100 μM beta amyloid. After 15 s of vortex, the tube was placed in 4 °C incubation for 24 h.

### Uptake of beta amyloid oligomers in cells

Different concentrations of Ab-T1 (0.5, 2, and 10 μg/ml) were added to cells with total 1 ml/well new culture media (RPMI 1640, 10% FBS, 2 mM l-glutamine, 1% Pen/Strep) and incubated at 37 °C for 8 h. Conditioned medium with PBS (without Ab-T1) was added to the cells as control. 0.3 μM beta amyloid oligomers (1-42) conjugated with Alexa 488 (oABeta-488) was added directly to cells in the culture plates and then cells were incubated 37 °C for additional 2 h before harvest (a number of wells were incubated without beta amyloid and Ab-T1 treatment as an additional control). Medium was removed, centrifuged, and saved for further analysis in ELISA assay. Extracellular and cell surface oABeta-488 in cell pellet was quenched by incubation of 0.4% Trypan blue in PBS (pH 4.4) for 1 min (adding directly in to the cells in the plates). Cells were washed and removed from culture plate by adding ice-cold PBS containing 2.5 mM EDTA, followed by incubation on ice for 30 min. Flow cytometry (CytoFLEX, Beckman coulter - FL1–blue laser (488 nm)) was used to measure beta amyloid oligomers (conjugated to Alexa Fluor 488) uptake indicated as relative geomean fluorescence intensity (gMFI).

### Uptake of apoptotic neurons

Human SH-SY5Y neuronal cells were stained using CFSE cell division tracker kit (BioLegend, Cat #: 423801) following manufacturer protocol. Stained SH-SY5Y cells were seeded (6 × 10^5^ cells/well) in a 12-well tissue culture plate and incubated overnight at 37 °C. In parallel, BV-2 microglia cells were seeded in two 10 cm tissue culture plates (7 × 10^6^ cells/plate), one of them incubated with Ab-T1 (5 μg/ml) and the second one with control IgG (BioLegend, Cat#: 403702) overnight at 37 °C. The next day, H_2_O_2_ (1.6 mM) was added to the stained neuronal cells for apoptosis induction (45 min at 37 °C). Activated and control microglia cells were added to the stained neuronal cells (1 × 10^6^ cells/well) for apoptotic cells uptake (1 h). Microglia cells in suspension were collected from each well (only the supernatant), washed and analyzed using flow cytometry (FL1–blue laser (488 nm) was used to measure neuronal CFSE uptake indicated as relative geomean fluorescence intensity (Relative MFI)).

### ELISA for assessment of TNF-alpha levels

TNF-alpha (TNFα) protein levels in cells were detected using DuoSet Elisa assay (R&D System) following manufacturer protocol.

### Lysate preparation

Cell supernatants, brain, and cell lysates were prepared in hypotonic buffer (0.01 M Tris, pH 7, 1 mM EDTA, 1 mM EGTA) freshly supplemented in protease inhibitor cocktail (P8340, Sigma)), incubated on ice for 30 min, snap frozen, thawed, followed by pellet resuspension in STE lysis buffer (150 mM NaCl, 50 mM Tis-HCl, pH 7.6, 2 mM EDTA, 1% Triton-X 100), and incubated on ice for 20 min before clarifying and supplemented with Laemmli sample buffer for SDS-PAGE protein separation.

### Microglia activation by Ab-T1 with Syk/Akt inhibitors

Mouse BV-2 microglia cells (ICLC cell bank, Cat No. ICLC ATL03001) were seeded (500,000 cells/well) in a 12-well tissue culture plate. Prior to studies with microglial activation and proliferation, the EC50 studies were conducted to determine the doses by which to conduct the main experiments. After 2 h, different concentrations of MK-2206 Akt inhibitor (ENZO, Cat: ENZ-CHM164-0005, 12 nM, 65 nM, 100 nM), R406 Syk inhibitor (BioVision, Cat #: 9682-5, 0.05, 0.1, 0.5, and 1 μM), or DMSO (control) were added and incubated with cells for 1 h at 37 °C. Two concentrations of Ab-T1 or control IgG (5 μg and 10 μg) were added to the cells for overnight incubation at 37 °C. The next day, supernatant and cells were saved in order to examine neuroinflammatory markers (TNFα and IL-1β levels) using Elisa or quantitative RT-PCR. Relative quantitation of gene expression was conducted by real-time PCR carried out using TaqMan® Gene Expression Assay (Applied Biosystems).

### The effect of Ab-T1 on Syk phosphorylation in BMDM

At the day of experiment, bone marrow-derived macrophages (BMDM) were harvested using Trypsin LE (ThermoFisher Scientific, Cat#: 12604054) and re-suspended in 10 ml starvation medium (1% FBS), for 4 h at 37 °C. Next, cells were divided into sample tubes (1.2 × 10^6^ cells/ml starvation medium). Five micrograms of Ab-T1 were added to samples for 2 or 5 min incubation on ice. For positive control, 0.1 mM of Na_3_VO_4_ (Enzo, Cat#: ALX-400-032-G025) together with 0.3 nM of H_2_O_2_ were added and cells were incubated at 37 °C for 10 min. After treatment, cells were spin down (2500×*g*, 10 min) and washed with cold PBS containing protease and phosphatase inhibitors (1 mM Na_3_VO_4_, 10 mM NaF (New England Biolabs, Cat#: P0759), and protease inhibitors cocktail (Sigma, P-8340, 1:100)). Cells were centrifuged (12,000 RPM, 5 min, 4 °C), aspirated with PBS, and lysed with full lysis buffer (150 mM NaCl, 25 mM Tris, pH 7.5, Triton, 0.5 mM EDTA, protease inhibitor cocktail, 1 mM Na_3_VO_4_, 10 mM NaF). Cell debris were removed by centrifuging samples at ~ 14,000×*g* for 15 min and kept on ice till immunoprecipitation (IP).

Lysates were immunoprecipitated with monoclonal antibody to mouse Syk (SYK-01, EXBIO, Cat #: 11-376-C100), 2 μg per sample bound to A/G PLUS-Agarose beads (SantaCruz Biotechnology, Cat #: sc-2003) following manufacturer’s instructions, then separated by SDS–PAGE protein separation on Nuphage 4-12% gels (ThermoFisher Scientific, Cat #: NP0322BOX) and transferred to Protran nitrocellulose membranes. Membranes were analyzed by immunoblot for phosphorylated Syk using mouse anti-Phosphotyrosine antibody (G410, Merck, Cat #: 05-321; 1:1000) following peroxidase-AffiniPure goat anti-mouse IgG (Jackson; 1:10,000) before enhanced chemiluminescence development (Fusion Solo 7S imager system, Vilber, France).

### Akt phosphorylation in activated BMDM

At the day of experiment, BMDM were harvested using Trypsin LE (ThermoFisher Scientific, Cat#: 12604054) and re-suspended in 10 ml starvation medium (1% FBS), for 4h at 37 °C. Next, cells were divided into sample tubes (3 × 10^6^ cells/ml starvation medium). Five micrograms of Ab-T1 or human control IgG (BioLegend, Cat#: 403702) were added to samples for 2 or 10 min incubation at 37 °C followed by cross linking with goat anti human Fc (Invitrogen, Cat#: H10500, 10 μg/ml) incubation at 37 °C for 10 min. After treatment, cells were spin down (2500×*g*, 10 min) and washed with cold PBS containing protease and phosphatase inhibitors (1 mM Na_3_VO_4_, 10 mM NaF, and protease inhibitors cocktail (Sigma, P-8340, 1:100)). Cells were centrifuged (12,000 RPM, 5 min, 4 °C), aspirated with PBS, and lysed with full lysis buffer (150 mM NaCl, 25 mM Tris, pH 7.5, Triton, 0.5 mM EDTA, protease inhibitor cocktail, 1 mM Na_3_VO_4_, 10 mM NaF). Cell debris were removed by centrifuging samples at ~ 14,000×*g* for 15 min and kept on ice till SDS-PAGE.

Twenty-five micrograms of lysates per sample were loaded in each well for SDS-PAGE protein separation on Nuphage 4–12% gels (ThermoFisher Scientific, Cat No. NP0322BOX). Samples were transferred to 0.2 μm nitrocellulose membranes (WhatmanProtan BA83, Cat No. 10401380) and incubated at 4 °C for 12 h with rabbit anti Phospho-Akt (Cell signaling, Cat #: 4060S; 1:2000) or Rabbit anti Akt (Cell signaling, Cat #: 9272S; 1:1000) as primary antibodies diluted in 5% BSA/TBST followed by goat anti mouse conjugated to HRP (Jackson, 1:10,000) for additional 1h incubation at room temperature. Membranes were developed using Fusion Solo 7S imager system (Vilber, France).

### Proliferation assay

Different concentrations of Ab-T1 and control IgG (2, 5, and 10 μg/ml) were added to BMDM cells (7 × 10^5^ cells/well) with total 1 ml/well new culture media and incubated at 37 °C for 72 h. Conditioned medium with PBS (without antibody treatment) was added to the cells as control. Cell proliferation was measured by flow cytometry (Quantifying cell population growth). Results are mean of four repeated experiments (3 wells for each sample per experiment).

### Regional distribution phenotype assessment of plaque-associated microglia

Tissues were fixed in 4% PFA, dehydrated in ethanol, and embedded in paraffin. Sequential double immunohistochemical staining was performed on 4-μm sections using the Leica BOND-MAX system (Leica Biosystems Newcastle Ltd, UK). Tissues were pretreated with epitope-retrieval solution (ER1, Leica Biosystems, Cat#: AR9961 Newcastle Ltd, UK) for 5 min followed by 30-min incubation with Beta-Amyloid (1:600, Abcam ab 2539) and Iba1 (Abcam, ab125212, 1:1500) primary antibodies (sequential double immunostaining). The Polymer Refine-HRP kit (Cat#: DS9800) for microglia staining and the Refine-Red kit (Cat#: DS9390) for beta amyloid plaque staining (Leica Biosystems Newcastle Ltd, UK) were used for enzymatic detection and tissues were counter-stained with Hematoxylin. An investigator blinded to the animal clinical data outlined the cortical (Primary motor cortex (M1) and S1HL) or hippocampal (CA1, CA2, CA3, and DG) region of interest on each slide using a brightfield auto cell imaging system (EVOS FL Auto Cell Imaging System, ThermoFisher Scientific). Twenty of 80 × 80 μm counting frames at × 20 magnification were randomly selected in each region (cortical or hippocampal) in which plaque associated microglia was counted by separate tags for stage I, II and III using ImageJ software to estimate the total number of microglia (stage I, II, and III) in the defined area. Different stages of activation were defined morphologically as previously described [1]. Data from four serial sections of tissue were averaged to obtain composite average densities of plaque-associated microglia in each region (average number of plaques in 20 frames from 4 serial sections (total of 80 frames in each region) in each brain sample from a group of mice). The number of plaques analyzed ranged from 100 to 150 in each brain.

The number of diffuse amyloid beta deposits (immunostaining with anti-amyloid beta, clone 4G8) in the cerebral cortex of 5xFAD mice was measured in three image frames per section at × 20 magnification (3 frames in 4 serial sections = total 12 frames in each brain sample). Images were captured using a digital slide scanner (Motic, China), converted to gray scale, and segmented with an auto threshold command (ImageJ).

### Thioflavin S staining for detection of beta amyloid plaques

Brains from 5xFAD mice were extracted and post-fixed in 4% PFA for 48 h and sunk in 30% sucrose. Brains were frozen on a cryotome platform and cut to generate 16- and 40-μm-thick sections. Sections were mounted on a glass slide and allowed to completely air dry prior to staining. Slides were washed with 70% ethanol (1 min) and 80% ethanol (1 min) followed by incubation with filtered thioflavin S (Sigma, Cat#: T1892) for 15 min in the dark. Thioflavin S stained slides were washed with 70% ethanol (1 min), 80% ethanol (1 min), and twice with distilled water before mounted in an aqueous mounting media. The green fluorescence stained plaques were visualized with fluorescent microscopy (EVOS FL Auto Cell Imaging System, ThermoFisher Scientific).

### Assessment of neuroinflammatory markers induced by intracerebral injection of soluble TREM2 in vivo

Twenty-four female (*n* = 3, 7, 7, 7) 5xFAD mice (12–14-week-old mice) were anesthetized by Isoflurane, and stereotaxically injected with human TREM2-ECD (3.6 μg of TREM-ECD per brain) in PBS with and without treatment (7.5 μg of Ab-T1 or hIgG4). Control sham C57BL/6JRccHsd 5xFAD animals received sterile PBS (*n* = 3). A single needle insertion (coordinates: − 2.2 mm relative to bregma, 2.0 mm from midline) into the right hemisphere was used to target the inoculum to the hippocampus located at a depth of 2 mm below the dura. Material was injected via a 10 μl Hamilton syringe at a rate of 0.4 μl per min (5 μl total volume) with the needle in place (33G) for ≥ 10 min at each target. Animals were inoculated at the right hemisphere unless otherwise indicated. Twenty-four hours post-intra hippocampal injections, animals were sacrificed, and brains were dissected and homogenized. In order to examine neuroinflammation, relative quantitation of gene expression was conducted by real-time PCR carried out using TaqMan® Gene Expression Assay (Applied Biosystems).

### The influence of treatment with Ab-T1 in the 5xFAD Alzheimer’s disease animal model

Thirty-seven female (4 groups of *n* = 9, 9, 10, 9) 5xFAD mice (4–5 months of age) were anesthetized by Isoflurane, intraperitoneal (i.p.) injected with anti-CD4 (clone GK1.5, InVivoMAb, BE0003-1) for CD4 cell depletion 6 days pre-treatment (experiment 1). Mice were treated i.p with humanized Ab-T1 (1 mg/kg and 10 mg/kg, *n* = 9, 10), human IgG (10 mg/kg, *n* = 9), and PBS as sham control (*n* = 9) once every 2 weeks for 3 months. Behavior tests (NOR and MWM) were conducted at various predetermined time points before they were sacrificed at 12 weeks post injections by overdose with ketamine/xylazine. For histological studies, the brain was removed and the left hemisphere underwent overnight postfixation with neutral-buffered formalin (Thermo Fisher Scientific), before being processed and embedded in O.C.T compound (Ref 4583, Tissue-Tek) for cryosectioning and immunostaining (Thioflavin S staining (Sigma, Cat#: T1892)) and immunohistochemistry. For biochemical studies, right hemisphere brain tissues were immediately frozen after removal and stored at − 80 °C until used. CSF and blood was taken from 20 animals (5 from each group). Blood was examined for hematological and biochemical parameters. TREM2 protein levels in CSF and serum were detected using mouse TREM2 sandwich ELISA kit (Cat#: LS-F7884, LifeSpanBioSciencesInc) following manufacturer protocol. Ab-T1 protein levels in brain and serum were detected using human immunoglobulin 4 (hu IgG4) sandwich ELISA kit (Invitrogen, Cat#: BMS2095). Amyloid beta protein levels in brain were detected using human amyloid beta 1-42 sandwich ELISA kit (Invitrogen, Cat#: KHB3544). In order to examine neuroinflammation, relative quantitation of gene expression was conducted by real-time PCR carried out using TaqMan® Gene Expression Assay (Applied Biosystems).

Experiment 2 was carried out in cognitively intact animals. Twenty-one female (3 groups of *n* = 7) 5xFAD mice (2 months of age) were anesthetized by Isoflurane, and intraperitoneal (i.p.) injected with anti-CD4 (clone GK1.5, InVivoMAb, Cat#: BE0003-1) for CD4 cell depletion 6 days pre-treatment. Mice were treated i.p with humanized Ab-T1 (0.5 mg/kg and 5 mg/kg) and PBS as sham control once a week for 2 months. Behavior tests (NOR and MWM) were conducted at various predetermined time points before they were sacrificed at 8 weeks from beginning of experiment by overdose with ketamine/xylazine followed by transcardial perfusion with PBS. For histological studies, the brain was removed and the right hemisphere underwent overnight postfixation with neutral buffered formalin (Thermo Fisher Scientific), before being processed and embedded in O.C.T compound (Ref 4583, Tissue-Tek). For biochemical studies, left hemisphere brain tissues were immediately frozen after removal and stored at − 80 °C until used.

### RNA purification and RT-PCR from brain tissue

Animals were sacrificed, and brains were dissected and homogenized. RNA was isolated using SV total RNA isolation system kit (Promega, Cat#: 20-410-100) following the manufacturer’s protocol. Complementary DNA (cDNA) synthesis was carried out with qScript cDNA Synthesis Kit (Quanta bio, Cat#: 95047) using 2 μg of total RNA as template. In order to examine neuroinflammation, relative quantitation of gene expression was conducted by real-time PCR carried out using TaqMan® Gene Expression Assay (Applied Biosystems, Cat#: 4369016). Fluorescent (FAM)-labeled IL-6, TNFα, INFγ, and IL-1b were normalized to an internal control, GAPDH. All comparisons refer to sham. Analysis was done using the Comparative Ct Method (ΔΔCT).

### Behavioral assessment

#### Novel object recognition

Twenty-four hours before testing, all animals were habituated to the testing apparatus for 10 min (50 cm box, 40 cm high). The day after, animals were introduced to the objects: first, two identical objects were placed in the box, and mice were allowed to explore objects for 5 min. The same procedure continued until 5 mice were done. The entire phase for 5 mice lasted 30 min. Immediately after, these 5 mice were tested in the same order as before. Animals were introduced to two different objects, one familiar object and one novel object that the mice never encountered. Mice were allowed to explore objects for 5 min and then removed from the box. At all phases, after each mouse was removed from the box, the box was sterilized with alcohol. Sample and novel objects and their locations were counterbalanced across animals. Each trial was videotaped, and time and frequency spent with each object was measured using NoldusEthoVision XT 11.5 (Noldus information Technology, The Netherlands). Memory was operationally defined by the discrimination index for the novel object (DI) as the proportion of time animals spent investigating the novel object minus proportion spent investigating the familiar one in the testing period [Discrimination Index, DI = (novel object exploration time − familiar object exploration time)/(novel exploration time + familiar object exploration)].

#### Morris water maze

The Morris water maze task requires mice to find a submerged platform in a large circular pool of water. This version of the task is a reference memory task where mice solve the task by learning the spatial relationships between the platform location and the extra-maze landmarks in the testing environment. The experimental apparatus consisted of a circular water tank (diameter = 100 cm; height = 40 cm) containing water at 22 °C to a depth of 32 cm. A platform (10 × 10 cm) was submerged 1 cm below the water surface and placed at the midpoint of one quadrant. The pool was placed in a test room homogenously brightened and containing various prominent cues. Mice were transferred from the housing facility to the behavior room at least 30 min before testing to adjust to the new environment. The swimming paths of the animals were recorded using a video tracking system. On day 1, navigation to a visible platform (The water was clear in the water tank and a flag was placed on the platform to increase visibility) was carried out to evaluate visual and motor abilities of animals and both the platform and starting direction change in each trial (pool was divided to 4 quadrants). Mice were gently placed into the water, facing the edge of the pool. If they failed to find the platform after 60 s, they were guided to its location and allowed to stay there for 20 s before returning them to their home cage. Mice that manage to find the platform were allowed to stay 5 s before their return. Mice were submitted to 5 trials per day with an inter-trial interval of at least 45 min. On days 2–5, the flag was removed from platform and aqueous acrylic emulsion paint was added to the water so that the submerged platform will not be visible from the surface of the water. Memory-acquisition trials (training) were performed five times daily with a 45-min inter-trial interval to reach a steady state of escape latency. The platform position remained constant while the starting direction changed. Mice failing to find the platform within 60 s were placed on the platform for 20 s at the end of the trial. On day 6, the platform was removed from the pool and the starting direction for the single trial was at the farthest (North) point from the platform quadrant used on days 2–5 (Southwest) so that the mice would travel some distance before entering the previously learned platform quadrant. One probe trial (60-s long) was performed for each animal. NoldusEthoVision XT 11.5 (Noldus information Technology, The Netherlands) software was used so that the camera can create physical distance information from pixel-based information after calibrating and defining parameters (dividing pool into 4 quadrants) in order to track path length, escape latency, and time spent in each quadrant.

Animal studies performed were blinded. Transgenic animals were randomly selected for treatment. Behavioral assessment data were analyzed identically (regardless of treatment selection) using NoldusEthoVision XT 11.5 software.

#### Statistical analysis

Values shown in the figures are presented as mean ± SEM unless otherwise mentioned. *P* values for determination of the statistical significance of differences were calculated by means of paired, two-tailed Student’s *t* test, two-tailed Mann-Whitney *U* test. Statistical analysis was performed using SPSS or Prism 8. *P* = 0.05 was defined as the level of significance. Unless otherwise indicated, in vitro data shown represent three independent experimental repeats with triplicate technical repetition.

## Results

### Ab-T1 recognizes TREM2 expressed on transfected HEK293T, stable U937 expressing cells, and exhibits a high affinity to human and mouse TREM2

A number of monoclonal antibodies against TREM2 were generated (hybridoma cells).

**Fig. 1:**
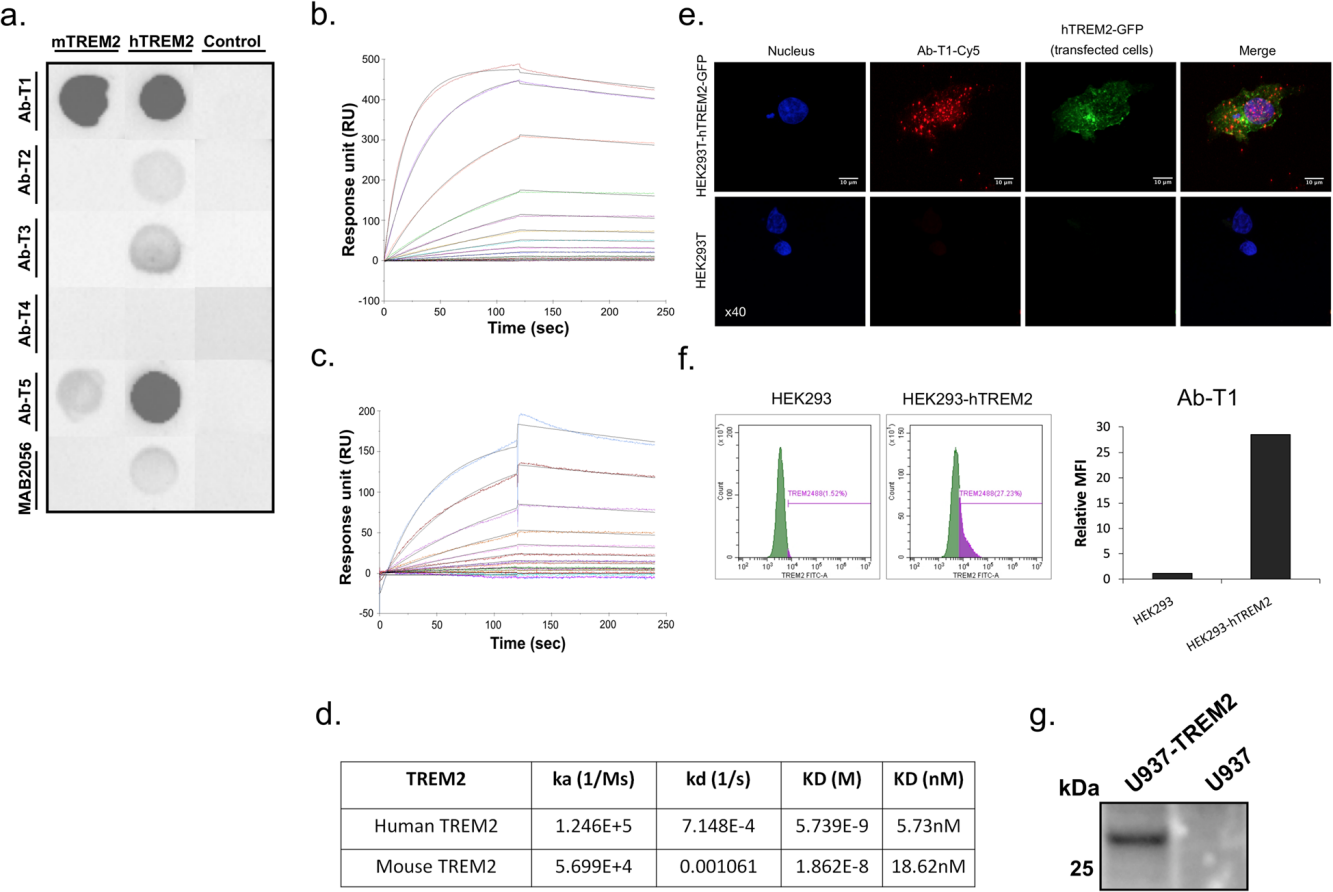
Binding of Ab-T1 to TREM2. **a** Binding of the mouse IgG antibodies Ab-T1, Ab-T2, Ab-T3, Ab-T4, Ab-T5, and control anti human TREM2 Ab to human and mouse TREM2 expressed in HEK293T cells by dot blot assay. Control naïve HEK293T cells (control) used as negative control. Binding affinity sensograms of Ab-T1 to (**b**) human TREM2 and (**c**) mouse TREM2 as measured by surface plasmon resonance (BiaCore). **d** Ab-T1 binding affinity table; “Ka” refers to the association rate constant; “kd” refers to the dissociation rate constant; and “KD” refers to the affinity constant. **e** Confocal images of Ab-T1 immunostaining non-permeabilised HEK293T transfected with wild type hTREM2-GFP (upper panels) and parental HEK293T (lower panels). Nuclei stained with DAPI. **f** Human TREM2 expression levels on transfected HEK293T cells recognized by Ab-T1. The *y*-axis represents the relative mean fluorescence intensity (relative MFI) measured by flow cytometry. **g** Western blot showing human TREM2 detection on stable U937 cell line recognized by Ab-T1

In order to evaluate the binding characteristics and affinity of monoclonal anti-TREM2 clones, cross-reactivity of mAbs with human TREM2 was confirmed by a number of immunoassays (Fig. 1a–d). Human embryonic kidney cells (HEK) were transfected with a DNA construct expressing human or mouse TREM2 (HEK-hTREM2-GFP or HEK-mTREM2-GFP) to evaluate mAbs binding to a full human or mouse TREM2 protein expressed on their surface. In HEK293T stable cells expressing TREM2, dot blots of the cell lysates were probed with different mAbs generated (Fig. 1a). Ab-T1 and Ab-T5 had the highest binding affinity compared to a commercial detection antibody to human TREM2 (Fig. 1a; MAB2056) while Ab-T1 showed the highest affinity to mouse TREM2. No binding was seen in naïve cells (Fig. 1a; control). Ab-T1 clone was further investigated to test its binding affinity in surface plasmon resonance (Biacore SPR system, GE Healthcare Life Sciences) (Fig. 1b, c) to human TREM2 extracellular domain (TREM2-ECD) and mouse TREM2-Fc conjugated (mouse TREM2). Ab-T1 binding affinity to human TREM2 (Fig. 1b, d; KD = 5.73 nM) and mouse TREM2 (Fig. 1c, d; KD = 18.63 nM). Ab-T1 was chosen for further testing. Non-permealised expressing hTREM2 HEK293T cells (Fig. 1e, f) and stably expressing hTREM2 U937 cells (Fig. 1g) showed high levels of TREM2 expression when probed with Ab-T1 monoclonal antibody. There was no TREM2 expression seen in HEK293T (Fig. 1e, f) and U937 (Fig. 1g) naïve cells probed with Ab-T1. No binding was evident to sTREM1 or TREML1.

### Ab-T1 recognizes soluble and membranous TREM2

**Fig. 2:**
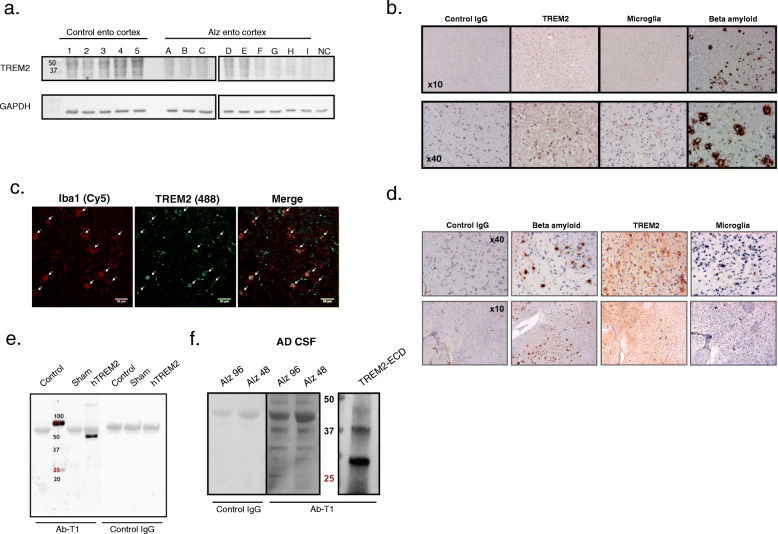
Recognition of TREM2 in AD models/human by Ab-T1. **a** Western blot showing Ab-T1 binding to human entorhinal cortex extracts from Alzheimer/control group patients. HEK293T protein lysate was used as negative control (NC). GAPDH as “housekeeping” protein loading control is shown in lower panels. **b** Immunohistochemistry staining showing Ab-T1 binding to human brain sample (entorhinal cortex sections) from Alzheimer’s disease patient (TREM2) with microglia and beta amyloid staining of same human brain tissue sample. Mouse IgG was used as negative control staining. **c** Confocal microscope scan images showing co-localization of TREM2 (mouse Ab-T1) with resident Iba1 positive cells (Microglia) in 5xFAD mice brain slices (white arrows). **d** Immunohistochemistry staining showing Ab-T1 binding to brain tissue from 5xFAD mice (TREM2) with microglia and beta amyloid staining of same mice tissue sample. Mouse IgG was used as negative control. **e** Western blots of supernatants (left panel) for soluble TREM2 detection in parental HEK293T vs. HEK293T-hTREM2 cells using mouse Ab-T1 and mouse IgG1 as control Ab. Transfected HEK293T cells with no insert DNA vector were used as sham. **f** Western blots of soluble hTREM2 detection in human CSF from Alzheimer patients using Ab-T1 and mouse IgG1 as control IgG antibody. TREM-ECD represents soluble TREM2 recombinant protein control

Ab-T1 was shown to bind its target TREM2 in a panel of entorhinal segments human brains (entorhinal cortex; ento cortex)) from Alzheimer’s disease patients and healthy subjects seen in Western blot (Fig. 2a). It can be clearly observed that several bands representing several TREM2 fragments are detected by the antibody representing soluble clipped forms in the extracellular space and internalized degraded form of the membranous protein. There was no binding to TREM2 in a negative control human tissue lysate which do not express TREM2 protein (Fig. 2a, NC).

### Ab-T1 robustly stains TREM2 in Alzheimer’s disease brains and co-localizes with resident microglia

To verify the binding of Ab-T1 evident in the Western blot, we used immunohistochemical studies employing the antibody. Ab-T1 stains TREM2 in human Alzheimer’s disease brain tissue samples seen by immunohistochemistry staining (Fig. 2b panel), demonstrating its ability to recognize TREM2 in multiple patients with Alzheimer’s disease and co-localize with resident microglia (Iba1; Microglia) near beta amyloid plaques in entorhinal cortex. As the IHC studies were performed on consecutive studies, we employed double immunofluorescence studies showing that TREM2 colocalizes with markers of microglia (panel 2c). To further confirm the relevance to the rodent models, Ab-T1 was shown to detect TREM2 in brains of 5xFAD Alzheimer’s disease mice (Fig. 2d) and co-localize with resident microglia (Fig. 2d; Microglia) and amyloid plaques (Fig. 2d; beta amyloid). No staining was seen with the control IgG.

### Ab-T1 binds soluble TREM2 in supernatants of TREM2 transfected cells and CSF of human Alzheimer’s disease patients

Soluble TREM2 was detected by Western blotting the conditioned medium from HEK293 cells transfected with TREM2 (Fig. 2e; hTREM2). Ab-T1 detected soluble TREM2 in supernatants of HEK293T cells transfected with human TREM2 (HEK293T-hTREM2) and not in naïve HEK293T cells (control) or sham control as shown in Western blots (Fig. 2e—Ab-T1 staining). Control mouse IgG antibody (control Ab) did not detect any soluble TREM2 forms in transfected HEK293T cells (Fig. 2e–control IgG). Human CSF from Alzheimer’s disease patients was blotted with Ab-T1 to determine its ability to bind endogenous soluble human TREM2. Ab-T1 detected soluble TREM2from AD patients CSF (Fig. 2f—Alz 93 and Alz 48, right panel) as compared to control IgG (Fig. 2f—Alz 93 and Alz 48, left panel). TREM2-ECD was used as positive control.

### Ab-T1 enhances acute microglial activation and promotes uptake of beta amyloid

**Fig. 3:**
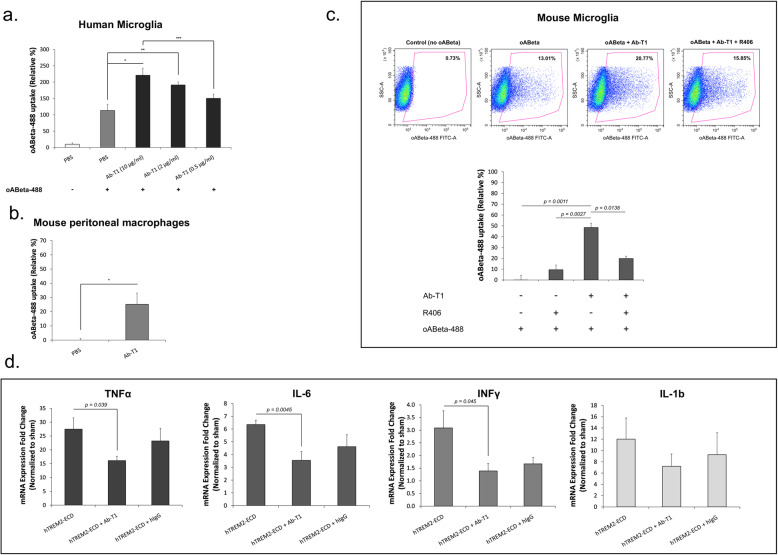
Ab-T1 augments uptake of beta amyloid in a Syk-dependent manner and reduces inflammation triggered by IC delivery of soluble TREM2. Ab-T1 increases uptake of labeled oligomeric beta amyloid **a** in human microglia derived from differentiated human PBMC’s, **b** murine peritoneal macrophages, and **c** murine microglia in a Syk dependent manner (R406). The *y*-axis represents the relative geometric mean (gMFI) measured by flow cytometry. Results are mean of three repeated experiments with duplicate technical sample repetition. **d** Intracerebral delivery of sTREM2 with Ab-T1 attenuates the neuroinflammatory response evident by delivery of sTREM2 with control IgG (*n* = 7, two-tailed student’s *t* test). The *y*-axis represents the fold-change in expression relative to sham (PBS injected animals)

Flow cytometry was used to measure beta amyloid oligomers conjugated to Alexa Fluor 488 (oABeta-488) cellular uptake (calculated by relative % of geomean fluorescent intensity (Relative %)) (Fig. 3a–c). Flow cytometry analysis shows that Ab-T1 increases human microglia like cells’ cellular uptake of oABeta conjugated to Alexa Fluor 488 in a dose-dependent manner (Ab-T1; 0.5 μg/ml: 140%, 2 μg/ml: 185% and 10 μg/ml: 205%) compared to cells with control treatment (PBS; 100%) seen in Fig. 3a. As shown in Fig. 3b, Ab-T1 increases mouse peritoneal macrophages cellular uptake (Ab-T1; 25.14 ± 7.94%) of oligomeric amyloid beta conjugated to Alexa Fluor 488 (oABeta-488) relative to sham treatment (PBS; 0 ± 1.09%, *P* = 0.039). Mouse microglia cells showed an increase in oAbeta-488 cellular uptake (Fig. 3c—Ab-T1; 48 ± 4.07%) relative to sham treatment (Fig. 3c–control; 0.02 ± 4.16%, *P* = 0.0011). Pre-incubation of microglia cells with Syk inhibitor (R406) attenuated ABeta-488 uptake (Fig. 3c—Ab-T1 + R406; 19.76 ± 2.22%, *P* = 0.0138) suggesting Syk phosphorylation is required for the uptake.

### Ab-T1 attenuates neuroinflammation induced by intracerebral injection of soluble TREM2 in vivo (suppression of chronic microglial activation)

It is known that CSF levels of sTREM2 are elevated in neuroinflammatory diseases as Alzheimer’s disease and multiple sclerosis [14] and potentially promote neuroinflammation [24]. Mice were injected with human-soluble TREM2 (TREM2-ECD) into the hippocampus (with and without Ab-T1 or hIgG treatment) aiming to test the effect of Ab-T1 on the neuroinflammatory response triggered by soluble TREM2. Ab-T1 downregulated a panel of pro-inflammatory cytokines that were induced in murine brains after 24 h of exposure to human TREM2-ECD (Fig. 3d). Figure 3d shows that Ab-T1 significantly downregulates TNF-α (*P* = 0.039), IL-6 (*P* = 0.0045), and INFγ (*P* = 0.045) mRNA expression levels after intracerebral injection of soluble TREM2 compared to no treatment (Fig. 3d; hTREM2-ECD; TNF-α: *P* = 0.039, IL-6: *P* = 0.0045, and INFγ: *P* = 0.045). There was no significant downregulation seen by the human IgG control (Fig. 3d; hTREM2-ECD + hIgG; TNF-α (*P* = 0.49), IL-6 (*P* = 0.14), and INFγ (*P* = 0.097)). Ab-T1 did not have an effect on IL-1b levels (Fig. 3d; IL-1b).

### Ab-T1 enhances activation of TREM2 expressing cells in a Syk-dependent manner

**Fig. 4:**
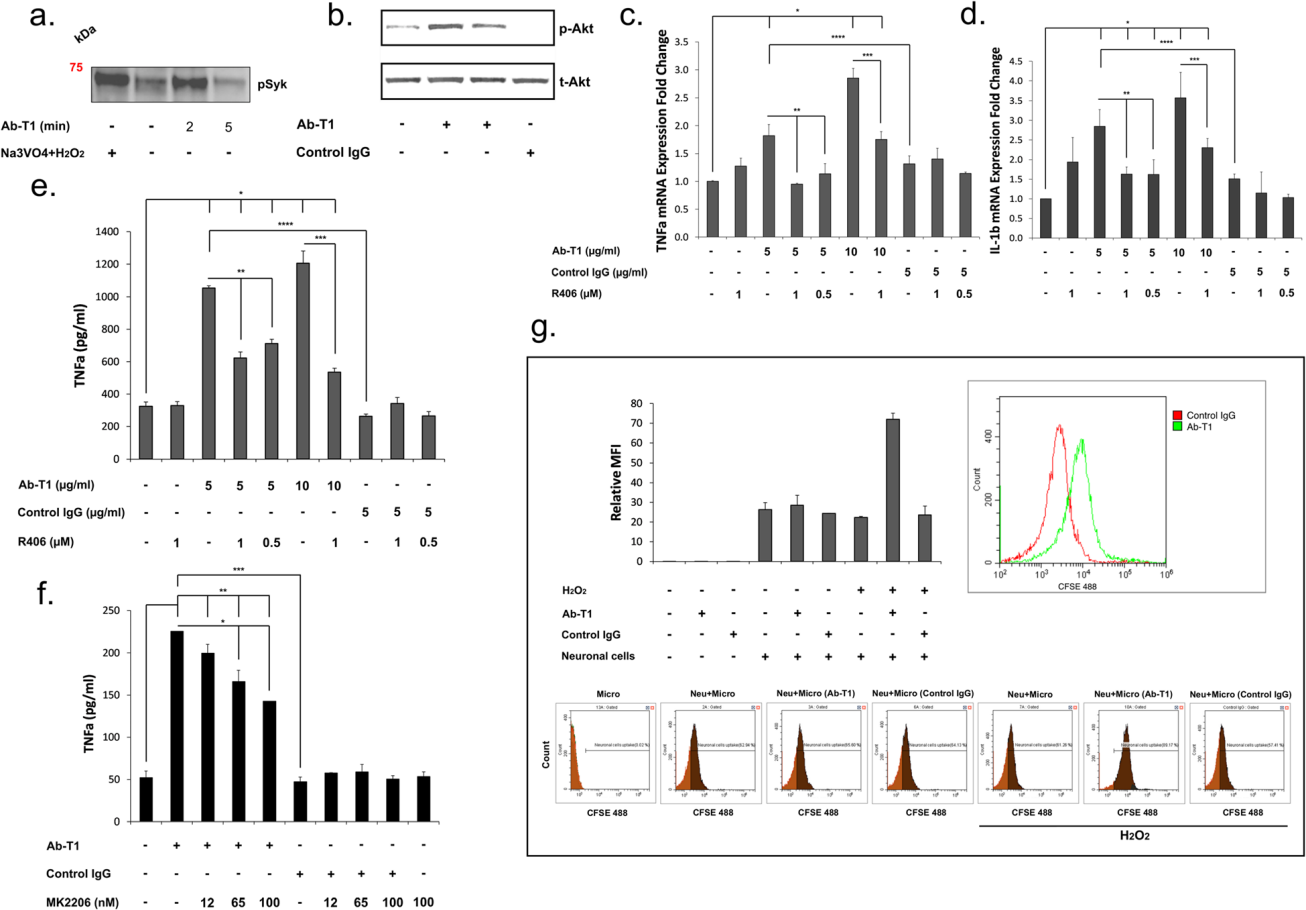
Effects of Ab-T1 on Syk/Akt phosphorylation and the activation of microglia and macrophages. **a** Ab-T1 promotes Syk phosphorylation and **b** Akt phosphorylation. Western blot analysis of phosphorylated Syk (Tyr525/526) or Akt (Ser473) in mouse bone marrow-derived macrophages stimulated with Ab-T1. Ab-T1 upregulates (**c**) TNFα and (**d**) IL-1b mRNA and TNFα protein levels in microglia dependent on **e** Syk or **f** Akt phosphorylation as the respective inhibitor attenuates consequent Ab-T1-induced activation. Data shown in ELISA assay represent triplicate repeats. **g** Ab-T1 enhances the uptake of labeled apoptotic neurons by microglia as compared to control IgG. Data shown represent triplicate experimental repeats with triplicate sample repetition

Ab-T1 activation of trem2 expressing cells was tested in a number of immunoassays (Fig. 4). In order to investigate if Ab-T1 induces tyrosine phosphorylation of cellular proteins such as Syk or Akt, TREM2 expressing bone marrow-derived macrophages (BMDM) were stimulated with Ab- with protease and phosphatase inhibitors before they were immunoprecipitated or blotted with an anti Syk or Akt antibody (Fig. 4a, b). Hydrogen peroxide with sodium orthovanadate (Fig. 3d; Na_3_VO_4_ + H_2_O_2_) used as positive control and Ab-T1 (Fig. 4a; Ab-T1) increased Syk phosphorylation in BMDM as seen in membrane blotted with anti-phosphotyrosine antibody. In a similar experiment, Fig. 4b shows induction of Akt phosphorylation in BMDM extracts seen in Western blot staining with anti-phosphorylated Akt and anti total Akt antibodies after 2 and 10 min of Ab-T1 stimulation. There was minimally detectable phosphorylated Akt protein detected in BMDM cells stimulated with control IgG or no treatment.

TNFα and IL-1β mRNA expression levels were measured in microglia cells treated with Ab-T1 or control IgG antibodies with and without spleen tyrosine kinase (SYK) inhibitor R406 (Fig. 4c, d). Microglia incubated with Ab-T1 exhibited more than a 3-fold increase in mRNA expression levels of TNFα and IL-1β (Fig. 4c, d) as compared to untreated cells. There was almost no increase in pro-inflammatory cytokines in microglia cells pre-incubated with R406 inhibitor before Ab-T1 treatment.

Ab-T1 concentrations (5 and 10 μg/ml) induced a 4-fold increase in TNFα protein levels in microglia cells as compared to control IgG or no treatment (Fig. 4e). There was no increase in TNFα protein levels in control IgG antibody-treated cells as compared to no treatment. Pre-incubation of Ab-T1 activated microglial in the presence of a Syk inhibitor R406, attenuated consequent activation evident by the lower TNFα protein levels in both 0.5 and 1 μM concentrations (Fig. 4e). R406 inhibitor alone did not have an effect in TNFα protein levels.

Additionally, TNFα protein levels were measured in microglia cells treated with Ab-T1 or control IgG antibodies with and without protein kinase B (AKT) inhibitor MK2206 (Fig. 4f). Ab-T1 increased more than 4-fold TNFα protein levels in microglia cells compared to control IgG and no treatment (Fig. 4f). Pre-incubation of microglia with the Akt inhibitor MK2206 attenuated Ab-T1 induced activation in a dose-dependent manner as seen by the lower TNFα protein levels with 12 nM, 65 nM, and 100 nM inhibitor concentration (Fig. 4f; 200, 166, 142 pg/ml respectively). MK2206 inhibitor alone did not have an effect on TNFα protein levels as compared to no treatment control.

### Microglia uptake of apoptotic neurons after Ab-T1 stimulation

As one of the important properties of microglia in ameliorating neurodegeneration is their ability to engulf and clear cellular debris. We thus tested the hypothesis that activation of microglia with Ab-T1 will be associated with an increased uptake of apoptotic neurons in vitro.

Stimulated microglia cells with Ab-T1 increased cellular uptake of CFSE-stained apoptotic neuronal cells (Fig. 4g; Neu + Micro (Ab-T1): relative MFI – 70) as compared to control IgG (Fig. 4g; Neu + Micro (control IgG): relative MFI – 20).

### Twice monthly dosing of Ab-T1 attenuates neuroinflammation and cognitive decline in female 5xFAD mice (experiment 1)

**Fig. 5:**
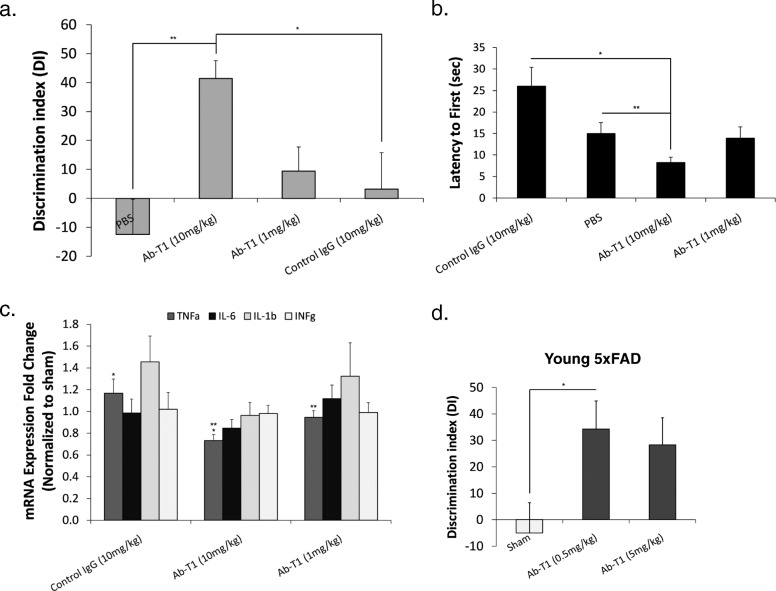
Ab-T1 improves cognition and attenuates neuroinflammation in AD 5xFAD Mice. **a** Five-month-old female mice treated with sham (PBS) and human IgG (10 mg/kg) showed cognitive impairment as indicated by the discrimination Index (DI) for the object recognition testing session (NOR) compared to Ab-T1 (10 mg/kg) treated mice (*n* = 9–10, two-tailed student’s *t* test, **P = 0.0019, *P = 0.0027) (**b**) and by the increase in latency to platform in the MWM probe test (two-tailed student’s *t* test, **P = 0.006, *P = 0.029). Error bars represent standard error of the mean. **c** TNFα, IL-1b, and IL-6 mRNA levels were detected using TaqMan real-time PCR in purified brain homogenates. The *y*-axis represents the fold-change in expression compared to no treatment (sham). GAPDH was used as “housekeeping” gene (two-tailed student’s *t* test, **P = 0.01, *P = 0.016). **d** Discrimination Index (DI) for the object recognition testing session (NOR) after 8 weeks of treatment dysfunction in young 5xFAD mice. (*n* = 7, two-tailed student’s *t* test, *P = 0.033). The result in the NOR can vary between + 100 and − 100, where a positive score indicates more time spent with the novel object, a negative score indicates more time spent with the familiar object, and a zero score indicates a null preference

5xFAD mice at 4–5 months of age, a time point where these mice are known to exhibit amyloid plaque burden within the brain, were intraperitoneally injected with different doses of Ab-T1 (1 and 10 mg/kg), a human IgG antibody (10 mg/kg) and PBS as sham control twice a month (*n* = 9–10 per group). A number of behavioral tests were conducted to examine their cognitive decline (Fig. 5a, b). Seven weeks post treatment, sham (Fig. 5a, PBS) and human IgG control-treated mice (Fig. 5a, hIgG; 10 mg/kg) exhibited significantly impaired novel object recognition compared to 10 mg/kg Ab-T1-treated animals (Fig. 5a; Ab-T1 (10 mg/kg)) as shown by the similar amount of time spent in exploring the two objects (familiar and new). There was no net preference between novel and familiar objects as shown in the reduced discrimination index [Discrimination Index, DI = (novel object exploration time − familiar object exploration time)/(novel exploration time + familiar object exploration) × 100]. Intraperitoneal treatment with Ab-T1 (Ab-T1 (10 mg/kg); DI 41.49 ± 6.13) significantly attenuated cognitive decline as compared to human IgG-treated animals (hIgG; DI 3.29 ± 12.49, *P* = 0.0027), 1 mg/kg dosage of Ab-T1 (DI 9.44) and untreated animals (PBS; DI 12.48 ± 12.22, *p* = 0.0019). Furthermore, 10 weeks post treatment, mice treated with 10 mg/kg of Ab-T1 showed a significantly shorter latency to escape onto the hidden platform in the Morris water maze probe test (Fig. 5b; 8.21 ± 1.27 s) as ompared to 10 mg/kg of human IgG (Fig. 5b; 25.97 ± 4.44 s, *P* = 0.029) and sham (Fig. 5b; PBS 14.94 ± 2.56, *P* = 0.006) treated control mice. There was no significant difference between human IgG control mice and 1 mg/kg Ab-T1-treated mice, similar to the novel object recognition test. In order to examine in vivo neuroinflammation, relative mRNA levels for a number of pro-inflammatory cytokines produced in the murine brain were quantified using RT-PCR 12 weeks post treatment (Fig. 5c). Ten milligram per kilogram Ab-T1 (Fig. 5c; 0.73 ± 0.057-fold change) downregulated TNFα expression levels relative to 10 mg/kg of human IgG control group (Fig. 5c; control IgG 1.17 ± 0.132-fold change, *P* = 0.016). There was no relative reduction in TNFα mRNA expression levels seen in 1 mg/kg Ab-T1 and human IgG control-treated mice. A similar trend was evident also with regard to IFN-g, IL-6, and IL-1b, although not statistically significant due to the relatively small number of samples.

### Weekly treatment with Ab-T1 effectively attenuates development of cognitive dysfunction in young 5xFAD mice (experiment 2)

After showing a therapeutic effect on mice with preexisting cognitive impairment, we went on to test the effects of Ab-T1 on the progression toward cognitive deterioration when treatment commences earlier at the presymptomatic stage.

Upon sacrifice, sham-treated mice exhibited significantly impaired novel object recognition compared to Ab-T1-treated animals as shown by the similar amount of time spent in exploring both familiar and novel objects (Fig. 5d; *P* = 0.033). There was no net preference between novel and familiar objects as shown in the reduced discrimination index [Discrimination Index, DI = (novel object exploration time − familiar object exploration time)/(novel exploration time + familiar object exploration) × 100]. Intraperitoneal treatment with Ab-T1 (Ab-T1 (0.5, 5 mg/kg); DI 34.2 ± 10.68, 28 ± 10.30) significantly attenuated cognitive decline as compared to untreated animals (sham; DI − 5 ± 11.44) (*n* = 7 per group).

### Intraperitoneal treatment with Ab-T1 reduces soluble amyloid beta as well as the number of amyloid plaques in aged male 5xFAD mice (experiment 1)

We used murine brains from experiment 1 to test the hypothesis that Ab-T1 would influence soluble amyloid beta levels as well as plaque numbers and size when initiated in aged plaque bearing 5xFAD mice [12].

**Fig. 6:**
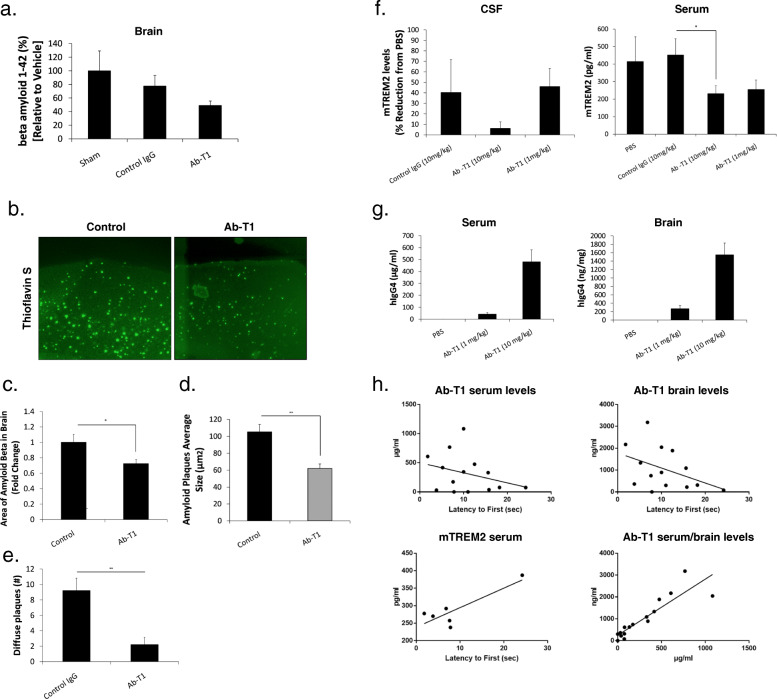
Effects on Ab-T1 on beta amyloid levels and target engagement in 5XFAD mice. **a** Ab-T1 treatment is associated with a decrease in total brain derived beta amyloid. **b** Beta amyloid plaques in mice brain sections were confirmed using thioflavin S fluorescent staining. **c, d** Images taken on microscope were analyzed using ImageJ software for mean number of plaques per mice brain section area and average plaque size (*n* = 9–10, two-tailed student’s *t* test, **P = 0.001, *P = 0.032). **e** Number of diffuse plaques in the cortex of Ab-T1 vs. control-treated 5xFAD mice (two-tailed student’s *t* test, **P < 0.005). **f** Ab-T1 induces a reduction of soluble TREM2 in CSF and serum (two-tailed student’s *t* test, *P = 0.047). **g** Ab-T1 levels in serum and brain of 5xFAD treated mice. (**h**; upper panels) Correlation of serum and brain levels of Ab-T1 with cognition tested in MWM (*r* = 0.46; *P* < 0.05 and *r* = 0.5; *P* < 0.05). (**h**; lower right) Association between serum and brain levels in the same treated mice (*r* = 0.89; *P* < 0.001). (**h**; lower left) Association of free sTREM2 levels achieved by Ab-T1 treatment, and cognition (*r* = 0.61; *P* < 0.05). Data shown in ELISA assays is the mean of triplicate sample repetition

Upon sacrifice, soluble amyloid beta levels in treated animals were reduced as compared to controls (Fig. 6a) but the effect was marginal and did not achieve statistical significance. Brain sections were subsequently stained with thioflavin S, which binds to the characteristic β-pleated sheet conformation [22]. Sections from mice treated with 10 mg/kg Ab-T1 (Fig. 6b and c; 0.72 ± 0.05) showed lower levels of plaques per total brain area compared to sham treated mice (Fig. 6b and c; 1 ± 0.10, *P* = 0.032, two-tailed *t* test). Image analysis of thioflavin S staining from same brain sections showed decrease in average plaque size in mice treated with 10 mg/kg Ab-T1 (Fig. 6d; 105.38 ± 5.16 μm, *P* = 0.0001) compared to sham-treated mice (Fig. 6d; 62.05 ± 8.84 μm, *P* = 0.0001, two-tailed *t* test). Brain sections were then immunostained with anti-beta amyloid antibody (clone 4G8) for diffuse plaque assessment. There was a significant decrease in the number of diffuse plaques in brain sections of Ab-T1-treated mice (Fig. 6e 9.2 ± 1.612; *P* = 0.0001) compared to sham-treated mice (2.2 ± 0.941, *P* = 0.0001).

### Ab-T1 dose dependently engages serum and CSF-free soluble TREM2

To quantify the levels of soluble TREM-2 (mTREM2) in experiment 1, in mice treated with Ab-T1 (experiment 1), an enzyme-linked immunosorbent assay (ELISA) directed against the extracellular portion of mouse TREM-2 protein was used (Fig. 6f). Soluble TREM2 levels were determined in all CSF and serum specimens collected from 5xFAD mice upon sacrifice. 5XFAD mice treated with Ab-T1 (10 mg/kg) exhibited significantly lower levels of sTREM2 in CSF (~ 95% reduction) compared to sham (PBS)-treated animals (Fig. 6f; CSF) suggesting a robust reduction of the soluble unbound TREM2 levels. Significantly lower levels of mouse sTREM2 (231.1 ± 45.19 pg/ml) were evident in serum of mice treated with 1 and 10 mg/kg of Ab-T1 compared to human IgG control (502.92 ± 99.91 pg/ml, *P* = 0.047) and sham (PBS; ~ 400 pg/ml) (Fig. 6f; serum). There was no significant reduction in sTREM2 CSF levels in animals treated with IgG control compared to PBS.

### Ab-T1 levels in experiment 1

Human IgG4 in brains and serum of animals 10 weeks post treatment with Ab-T1 (1 or 10 mg/kg twice a month) were measured (Fig. 6g). Average serum levels of 10 mg/kg Ab-T1-treated animals were ~ 10 times higher (482 μg/ml) than 1 mg/kg treated animals (42.7 μg/ml). Brain levels of 10 mg/kg Ab-T1-treated animals were ~ 6 times higher than 1 mg/kg treated animals (270 ng/ml). There was a significant correlation between serum and brain levels of Ab-T1.

Importantly, serum and brain Ab-T1 levels were positively correlated with the improvement in cognition evident in the Morris water maze (MWM) test (Fig. 6h; *r* = 0.46; *P* < 0.05 and *r* = 0.5; *P* < 0.05, respectively). Moreover, it was observed that the lower the sTREM levels as a measure of engagement by Ab-T1, the better the cognition was (Fig. 6h lower left: *r* = 0.61; *P* < 0.05). Serum and brain levels of Ab-T1 were highly correlated (*r* = 0.89; *P* < 0.001; Fig. 6h lower right) suggesting a reproducible access of the antibody through the blood brain barrier.

### Regional distribution phenotype assessment of plaque associated microglia

**Fig. 7:**
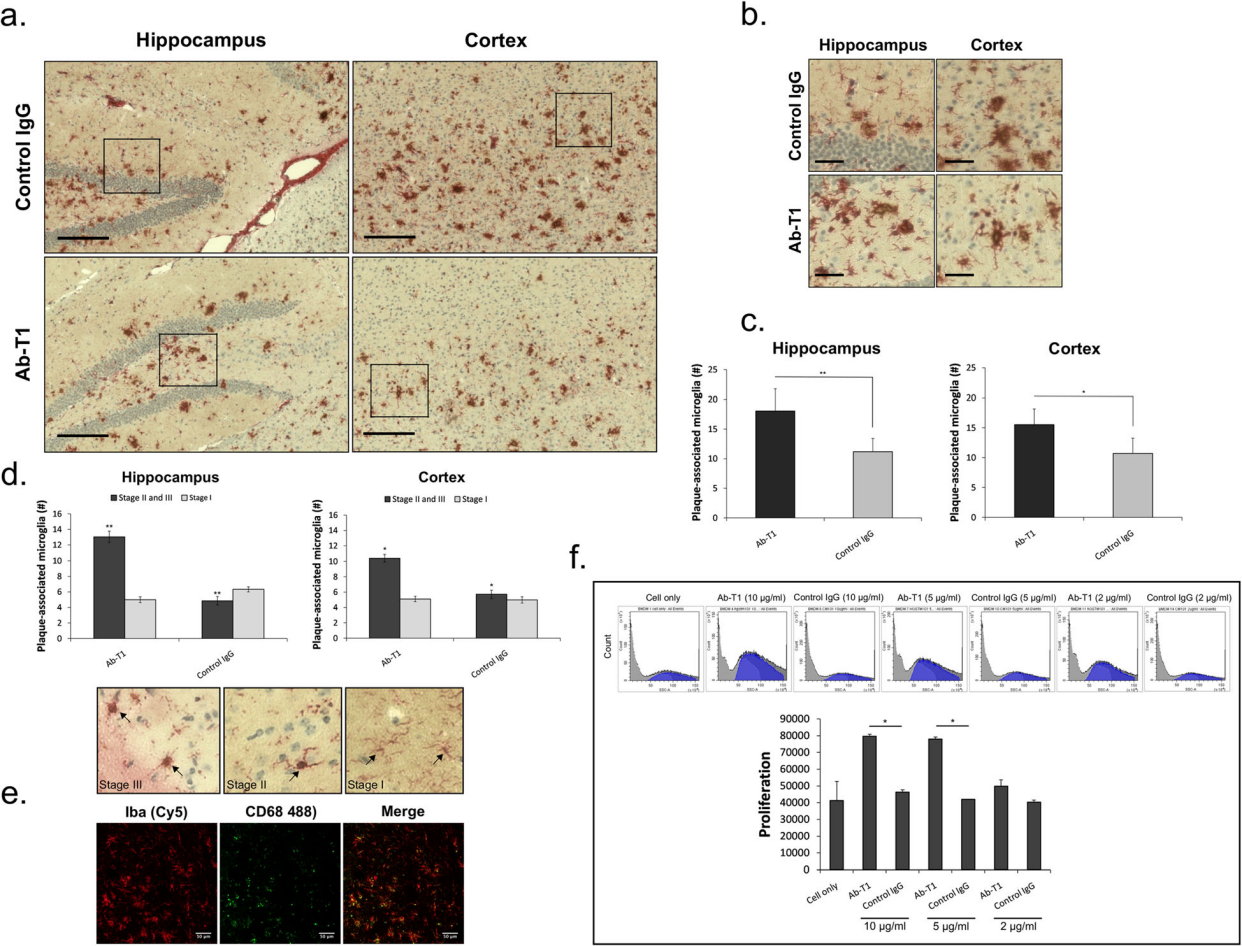
The effects of Ab-T1 on the number and activation status of plaque associated microglia**.** Ab-T1 induces an increase in the number of microglia activated in the vicinity of the plaques. **a** Hippocampus and cortex region representative micrographs of brain section of 5xFAD Alzheimer’s disease mice treated with Ab-T1 and control IgG (experiment 1). Black squares indicate an example of randomly selected 80 × 80 μm counting frame at × 20 magnification. **b** Enlarged images (× 20) of randomly selected frames from representative micrographs. **c** The average number of plaque associated-microglia in the hippocampus and cortex regions of treated mice. **d** A more efficient plaque coverage by a higher number of in situ associated microglia (average plaque-associated microglia; upper panels) based on different stages of microglia activation defined by their morphology (activated microglia—stage II and III) illustrated in the lower panels. (**c**, two-tailed student’s *t* test, **P = 0.0006, *P = 0.00024), (**d**, two-tailed student’s *t* test, *P = 0.0000028, **P = 0.00000065). Scale bar = 200 μm (**a**) and 50 μm (**b**). The immunohistochemistry studies were conducted employing double staining with anti-beta amyloid (4G8; red) and anti-Iba1 (brown) antibodies. **e** Confocal microscope images showing co-localization of CD68 with Iba1 positive cells in 5xFAD mice brain slices. **f** Ab-T1 but not control IgG augments BMDM induced proliferation. Cell proliferation was measured by flow cytometry (quantifying cell population growth). Results are mean of four repeated experiments with triplicate technical sample repetition

Cortical and hippocampal regions in a number of consecutive sagittal brain slices of treated (Ab-T1 or control IgG) 5xFAD animals (experiment 1) were analyzed and quantified for plaque-associated microglia. An investigator blinded to the animal clinical data quantified different stages of microglia (stage I, II, and III) to estimate the total number of plaque-associated microglia and activated microglia in each region [1] (Fig. 7). Double immunostaining images of beta amyloid plaques and microglia in both hippocampal and cortical regions show higher number of plaque-associated microglia in Ab-T1-treated animals compared to IgG-treated animals in proximity to the reduced plaques (Fig. 7a). Plaque-related activated microglia were quantified using magnified images (20 frames in each region per slice) (Fig. 7b). There were significantly higher number of plaque-associated microglia in Ab-T1-treated animals as compare to IgG-treated animals in both hippocampal and cortical regions (Fig. 7c, **P = 0.0006, *P = 0.00024, respectively). Furthermore, Fig. 7d shows that there were higher number of plaque-associated activated microglia (stage II and III) in Ab-T1-treated animals as compared to control IgG-treated animals in both hippocampal and cortical regions (cortex *P* = 0.0000028, hippocampus *P* = 0.00000065). The number of stage I microglia in both the hippocampus and cortex were the same as seen by the light grey bar in Fig. 7d. To further confirm the activation status of plaque-associated microglia in mice treated with Ab-T1, we stained respective brain sections with an antibody to CD68 (a lysosomal protein abundantly present activated microglia). Indeed, costaining with Iba-1 and CD68 confirmed the activation status of plaque-associated microglia in treated animals (Fig. 7e).

### Ab-T1 enhances proliferation of TREM2 expressing cells

Next, we wished to support the IHC data showing activated plaque-associated microglia and confirm the ability of Ab-T1 to induce proliferation in an in vitro system. Figure 7f shows dose-dependent cell proliferation in BMDM stimulated with Ab-T1 in 3 different concentrations (2, 5, and 10 μg/ml) as compared to control IgG and no treatment.

## Discussion

TREM2 loss of function mutations and variants are associated with a higher risk of AD. This observation has fueled the idea that this immune receptor expressed in microglia can play a role in the complex pathogenesis of AD. Neuroinflammation appears to have opposing roles in pathogenesis of neurodegeneration. Whereas acute, contained innate immune activation is protective by attempting to compact and limit plaque size, ongoing activation results in secretion of a panel of proinflammatory cytokines inducing a plaque-associated hostile environment that acts to promote neurodegeneration. Herein, we describe the production and testing of a novel antibody that addresses both pathogenic processes, namely induction of protective acute microglial activation while tuning down detrimental chronic neuroinflammation.

Ab-T1 was raised against the ECD of TREM2 aiming to avidly engage it to produce an activating signal. Importantly, the antibody selected for humanization was highly cross-reactive with murine TREM2 so as to provide a valid therapeutic agent in the genetically driven murine AD models, where it would engage murine microglial TREM2. We have observed that the antibody clearly recognizes TREM2 on transfected cells and in PBMC-derived microglia as well as in murine macrophages. More importantly, in brain sections from AD, Ab-T1 has been clearly shown to identify TREM2 expressed in resident microglia. Interestingly, Ab-T1exhibited intracellular staining in microglia that was also evident in the extracellular space suggesting the shed forms of TREM2 that are either clipped extracellularly or intracellularly and are bound and detectable by the antibody. We have also noticed that the expression of all TREM2 forms appeared to be reduced in AD brains. This observation may be related to the late “burnt out” stage of the disease, in those post mortem-obtained brains, where neuroinflammation is known to be significantly reduced as compared with early AD and MCI stages. Importantly, Ab-T1 also clearly binds to sTREM2 cell-free CSF of AD patients and its effects in vivo may be also related to this property.

Herein, we have shown that Ab-T1 activates TREM2 as evident by its ability to enhance uptake of labeled beta amyloid by microglia and macrophages and to promote TNF alpha production. These functions were dependent on Syk and Akt phosphorylation as their respective inhibitors ameliorated both these effects. Importantly, not only beta amyloid uptake was promoted by Ab-T1 but also the ability of microglia to engulf labeled apoptotic neurons representing a potential cellular debris within the hostile plaque environment.

Aiming to study how Ab-T1 influences chronic neuroinflammation, we have employed the short-term model previously reported by intracerebrally injecting soluble TREM2. We observed that co injection of Ab-T1 with sTREM2 attenuated brain proinflammatory cytokine mRNA levels. If indeed sTREM2 plays a role in driving chronic neuroinflammation as potentially evident circumstantially by its higher levels in the CSF of patients with early AD, binding and neutralization of sTREM could potentially be expected to attenuate neurodegeneration in vivo.

We next tested whether systemic treatment with Ab-T1 would influence the cognitive phenotype in various AD models. When tested after cognitive deficits were already present, in 7-month-old 5xFAD mice, Ab-T1 was also effective in attenuating cognitive impairment. These effects were associated with reduced neuroinflammation evident by reduced mRNA levels of a panel of neuroinflammatory cytokines and reduced amyloid plaque numbers which was cross-verified by quantitative levels of beta amyloid in the respective cortical fractions. We have also shown that proliferation of TREM2 expressing cells is significantly enhanced by exposure to Ab-T1. This finding was evident in the immunohistochemical studies showing a higher number of plaque-associated microglia in the antibody treated animals and the in vitro studies showing an isolated proliferative effect of Ab-T1 on BMDM. Notably, not only the number of plaque-associated microglia was increased in the treated animals but also they were displaying an active phenotype by their morphology and expression of CD68. Collectively, this series of studies suggest that Ab-T1 activates microglia and enhance their capacity to serve as potential mediators of plaque degradation, insulation, and compaction. In accord with the in vitro tests, analysis of plaques in the 5xFAD mice treated with Ab-T1 disclosed a significantly more effective plaque coverage by activated microglia.

Analysis of Ab-T1 concentrations in the sera, CSF, and brain of treated 5XFAD mice indicated that at 10 mg/kg, approximately 1% of serum concentrations were available for intracerebral TREM2 engagement in the extracellular cortical space. Moreover, target engagement shown by dose-dependent reduction in murine serum and CSF sTREM levels is supportive of the hypothesis that neutralization sTREM or reduction of its free levels would attenuate consequent chronic neuroinflammation. Indeed, the higher the levels of Ab-T1 were correlated with lower free CSF-derived sTREM2 levels and improved cognitive function.

Ab-T1 treatment was also effective in attenuating development of cognitive impairment in young cognitively intact and plaque free 5xFAD mice, suggesting a potential beneficial role in prevention and treatment.

Conflicting data has been obtained with regard to the effect of TREM2 gene deletion on cognitive deficits and amyloid plaque size in murine models of AD. These are probably the result of different timings of testing behavioral functions. We have been able to observe that regardless of whether mice were cognitively competent or compromised, treatment with Ab-T1 was effective, suggesting that knocking out TREM2 would not necessarily result in an opposite effect to TREM2 engagement and activation that was clearly beneficial in vivo.

Collectively, the data in the paper suggests that the mechanism of action of Ab-T1 is dual: enhancing and potentiating short-term microglial activation evident by the series of in vitro tests and attenuation of chronic neuroinflammation by antagonizing proinflammatory soluble TREM2 evident by its respective brain level reduction and the antibody’s effect in downregulating soluble TREM2-mediated neuroinflammation.

## Conclusion

We developed a mAb engaging the ECD domain of TREM2 that has been shown to attenuate cognitive impairment in amyloidopathy models. These beneficial effects are possibly related to the ability of Ab-T1 to promote acute microglial activation while concomitantly attenuating chronic neuroinflammation. Thus, TREM2 engagement and activation may represent a promising therapeutic modality in patients with AD.

## Data Availability

All data are included.

## Abbreviations

TREM: Triggering receptor expressed in myeloid cells
sTREM: Soluble TREM
ECD: Extracellular domain
mAb: Monoclonal antibody
AD: Alzheimer’s disease
ELISA: Enzyme-linked immunosorbent assay
BMDM: Bone marrow-derived macrophages
PBMC: Peripheral blood mononuclear cells
NOR: Novel object recognition
MWM: Morris water maze
TNF: Tumor necrosis factor
IgG: Immunoglobulin
NHD: Nasu-Hakola disease
